# Design and Experimental Study of Octopus-Inspired Soft Underwater Robot with Integrated Walking and Swimming Modes

**DOI:** 10.3390/biomimetics11010059

**Published:** 2026-01-09

**Authors:** Xudong Dai, Xiaoni Chi, Liwei Pan, Hongkun Zhou, Qiuxuan Wu, Zhiyuan Hu, Jian Wang

**Affiliations:** 1 HUD-ITMO Joint Institute, Hangzhou Dianzi University, Hangzhou 310018, China; 2 GEELY Automative Institute, Hangzhou Vocational & Technology University, Hangzhou 310018, China; 3 School of Automation, Hangzhou Dianzi University, Hangzhou 310018, China; 4 Kunming Shipborne Equipment Research and Test Center, Kunming 655211, China

**Keywords:** biomimetic underwater robot, soft robotics, octopus-inspired locomotion, bipedal walking, multi-arm swimming, gait control, rigid–flexible hybrid mechanism

## Abstract

To enhance the flexibility and adaptability of underwater robots in complex environments, this paper designs an octopus-inspired soft underwater robot capable of both bipedal walking and multi-arm swimming. The robot features a rigid–flexible coupling structure consisting of a head module and eight rope-driven soft tentacles and integrates buoyancy adjustment and center-of-gravity balancing systems to achieve stable posture control in both motion modes. Based on the octopus’s bipedal walking and multi-arm swimming mechanisms, this study formulates gait generation strategies for each mode. In walking mode, the robot achieves underwater linear movement, turning, and in-place rotation through coordinated tentacle actuation; in swimming mode, flexible three-dimensional propulsion is realized via synchronous undulatory gaits. Experimental results demonstrate the robot’s peak thrust of 14.1 N, average swimming speed of 8.6 cm/s, and maximum speed of 15.1 cm/s, validating the effectiveness of the proposed structure and motion control strategies. This research platform offers a promising solution for adaptive movement and exploration in unstructured underwater environments.

## 1. Introduction

Underwater soft robots have attracted increasing attention in recent years due to their outstanding compliance, adaptability, and environmental interaction capabilities, which make them particularly suitable for complex underwater tasks such as exploration, inspection, and manipulation [1]. Among marine organisms, cephalopods—especially octopuses—have become a key source of inspiration for the design of multi-modal soft robots, owing to their versatile locomotion and manipulation abilities. Octopuses exhibit a wide variety of locomotion modes, including jet propulsion, free swimming, crawling, and bipedal walking [2]. Moreover, their soft, highly dexterous arms enable grasping, anchoring, and manipulation, allowing them to adapt seamlessly to diverse environmental conditions.

Drawing inspiration from these biological characteristics, numerous octopus-inspired soft robotic systems have been proposed in recent years [3,4,5,6,7,8,9,10,11]. These systems primarily replicate one of the octopus’s locomotion patterns—such as crawling, jet propulsion, or multi-arm swimming—to perform specific underwater operations. However, most existing studies are limited to a single locomotion mode, lacking the ability to combine multiple types of movements within a single robotic platform.

Currently, the PoseiDRONE robot developed by the Italian Institute of BioRobotics represents one of the few attempts to integrate both swimming and walking functions in an octopus-inspired robot [12]. This system employs a crank–rocker mechanism for walking and a jet propulsion unit for swimming. Nevertheless, due to its rigid mechanical configuration and propulsion limitations, the robot can only generate fixed-directional thrust during walking and forward-only motion during swimming. Furthermore, it relies on an external power supply, which restricts its autonomy and operational flexibility. These limitations highlight the need for further research into fully self-contained, multi-modal soft robots that can achieve efficient walking and swimming through flexible, cable-driven actuation and coordinated gait control.

To overcome the limitations of existing underwater robots in adapting to rugged terrain, minimizing seabed disturbance, and enhancing multifunctionality, this study proposes a novel octopus-inspired soft-bodied underwater robot equipped with eight flexible rope-driven limbs. Inspired by octopus bipedal locomotion [10] and synchronous multi-arm propulsion [12], the robot can perform multi-directional crawling on the seabed and multi-arm swimming. Based on the kinematic characteristics of these two biological movement modes, this paper develops a gait generation strategy to achieve smooth transitions between crawling and swimming modes. Additionally, a series of underwater experiments evaluated the robot’s straight-line crawling, turning, and swimming performance. The proposed design lays the foundation for the development of adaptive, low-disturbance, and multifunctional soft-bodied underwater robotic systems capable of performing tasks in unstructured marine environments. These observations on octopus bipedal locomotion provide a biological basis for the gait design of the proposed robot.

## 2. Design of the Bionic Octopus Underwater Soft Robot

**Figure 1:**
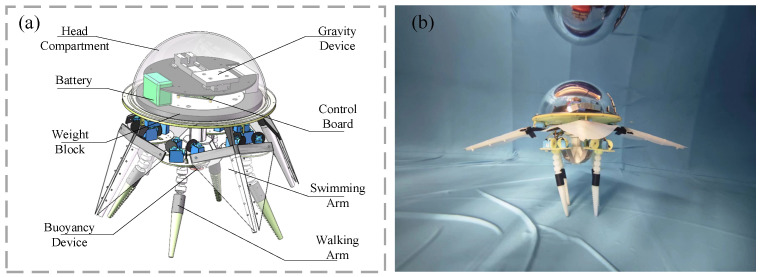
(**a**) Three-dimensional schematic of the bionic octopus-inspired robot. (**b**) Actual underwater bionic octopus-inspired robot.

The bionic octopus robot is designed using a rigid–flexible coupling approach. Its overall structure mainly consists of a head compartment and eight flexible biomimetic tentacles, as shown in Figure 1. The robot has a total mass of 8.12 kg in air, with physical dimensions of 500 mm × 350 mm × 350 mm, and the total length of the bionic arms is 230 mm. The arm segments are made from flexible materials to enhance their compliance on the seafloor. The robot platform is energy-autonomous, powered by an onboard battery, and can operate continuously for approximately 1 h. The main components are described as followed.

### 2.1. Head Compartment

The robot’s head compartment is primarily used to house non-waterproof components such as sensors, power supplies, and control boards. The head compartment consists of a transparent acrylic hemispherical dome and a 3D-printed circular base. The acrylic hemispherical dome is semi-spherical, with a diameter of 300 mm. The circular base has 7 holes on the bottom. The central hole is used for routing the signal and power cables of the buoyancy adjustment device, while the signal and power cables for the external drive arms can be connected to the internal control board through four square holes. The remaining two holes are used for mounting the depth sensor and the robot’s power switch. The acrylic hemispherical dome and the circular base are sealed using an O-ring (FKM) and secured with 12 screws. Additionally, extra ballast weight blocks are fixed onto the circular base of the robot’s head compartment to achieve neutral buoyancy. The robot’s center of buoyancy is designed to be above its center of gravity, providing the robot with passive stability.

### 2.2. Biomimetic Arms

Based on the need for multi-modal movement and operation of the robot, the eight biomimetic arms are designed as two types: walking drive arms and swimming drive arms. The walking drive arms are primarily used for the biomimetic robot’s walking and grasping functions, while the swimming drive arms are designed to enable multi-arm swimming.

**Figure 2:**
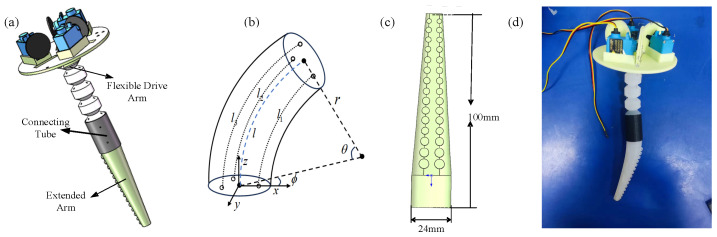
Structural design of the walking arms of the biomimetic octopus robot. (**a**) Three-dimensional walking drive arm. (**b**) Schematic of simplified flexible actuated arm. (**c**) Extended arm. (**d**) Walking drive arm.

Octopus bipedal walking or running primarily relies on the alternating propulsion of a pair of octopus arms. During this process, the arms continuously transition between bending and straightening states. To achieve the bending motion of an octopus’s arm, the octopus needs to contract its longitudinal muscles and slightly contract its transverse muscles. This mechanism can be simplified to the expansion and contraction of longitudinal actuators. In the biomimetic robot, the bending of the walking arms is primarily achieved through flexible cable-driven arms [9], as shown in Figure 2a,d. The structure of the walking arm is divided into three parts: The first part is the flexible drive arm, located at the base of the walking arm. It uses a three-cable drive system and can bend up to 360°, providing both bending and support functions for the biomimetic robot’s walking arm. Each walking (flexible drive) arm is tendon-driven by three cables (*l*_1_, *l*_2_, and *l*_3_) routed through three guide holes evenly distributed at 120° around the arm cross-section in Figure 2b. The cables are anchored at the arm tip and guided through the base support. By differentially shortening one cable with respect to the other two, the arm bends toward the shortened cable; by coordinating the three cable lengths, the arm can also be straightened or driven to a target bending plane and curvature. A schematic of the three-cable routing and the resulting bending/straightening mechanism is provided in Figure 2b. The second part is the connecting tube, which links the flexible drive arm with the extension arm. The connecting tube contains eight symmetrically placed pin holes, into which rigid pins are inserted and secured with silicone. The third part is the extended arm, which is cone-shaped. It connects to the end of the flexible drive arm, with a length of 100 mm and a terminal radius of 12 mm. The arm is equipped with two rows of small suction cups, totaling 30 cups, with 15 on each side, as shown Figure 2c. The largest suction cups have a diameter of 6 mm, while the smallest have a diameter of 2.45 mm. The suction cups are cylindrical in shape, with a hemispherical concavity at the center of the bottom. Their primary purpose is to increase the frictional force of the robot during walking.

The preparation of the silica gel drive arm follows the “pouring curing” process. The two-component RTV silica gel is mixed with an equal mass ratio (a:b = 1:1) and poured into the 3D printing mold. Three stainless steel rods (as inserts) are placed in the mold to reserve through holes for cable shuttling. Before curing, the mold filled with silica gel is vacuum-degassed to eliminate internal bubbles. The mold is then cured in an oven at 45 °C for 4–5 h. In order to improve the molding integrity and demolding reliability, the drive arm is poured symmetrically in two halves, then bonded and cured again with a/b silicone mixed glue. After complete curing, the stainless steel rod is carefully pulled out, the demolding of the drive arm is completed, and the excess overflow (flash) is trimmed.

**Figure 3:**
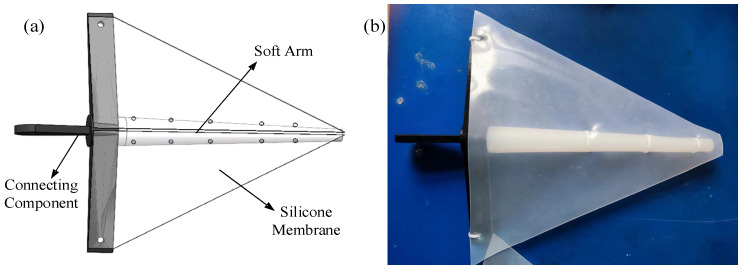
Structural design of the swimming drive arms of the bionic octopus robot. (**a**) Three-dimensional swimming drive arm. (**b**) Swimming drive arm.

The robot features four swimming drive arms symmetrically mounted at the rear of the head compartment, as shown in Figure 3. The soft arms are conical in shape, with a length of 200 mm. The end cross-sectional radius is 10 mm, while the top cross-sectional radius is 5 mm. The fabrication of the soft arms follows the same method used for producing the silicone arm in the flexible drive arm. Each swimming drive arm is equipped with a waterproof servo motor, which allows for a 90° range of rotation. The motor drives the rotation and swinging of the arms, providing the necessary propulsion for multi-armed swimming. To enhance hydrodynamic efficiency during swimming, triangular silicone membranes are added to the swimming drive arms, mimicking the webbing found between an octopus’s arms. These membranes are made from cut silicone and are secured to the swimming drive arms using zip ties.

### 2.3. Buoyancy Device and Center-of-Gravity Adjustment Device

**Figure 4:**
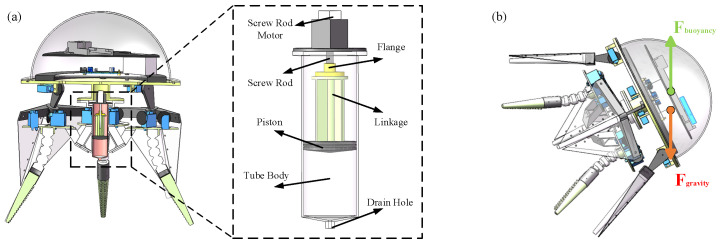
(**a**) Buoyancy adjustment device of the biomimetic octopus robot. (**b**) Robot tilting under the influence of the center-of-gravity adjustment device.

To ensure that the biomimetic octopus robot achieves a negative buoyancy state in the underwater walking mode and maintains neutral or positive buoyancy during swimming mode, a buoyancy adjustment device is embedded in the cavity of the robot’s connecting frame (designed to couple the head compartment with the four walking drive arms) to regulate its buoyancy. Figure 4a illustrates the structure of the buoyancy adjustment device. To accommodate the length constraints of the connecting frame cavity, the buoyancy device integrates a screw rod within the tubular body of the device and uses a piston-type structure.

The buoyancy device uses a screw motor as the power source. It drives a piston in reciprocating motion via a flange and connecting rod to perform the operations of pumping and draining, thereby adjusting the robot’s buoyancy. When it is necessary to increase the robot’s buoyancy, the screw motor drives the piston to expel water, reducing the liquid in the cavity. Conversely, when it is necessary to decrease the robot’s buoyancy, the screw motor drives the piston to intake water, increasing the liquid in the cavity.

Additionally, to enable the bionic octopus robot to perform upright walking and inclined swimming, a center-of-gravity adjustment device is added to the head compartment. The center-of-gravity adjustment device mainly consists of a ballast weight, a guide rail, and a screw motor. It allows the ballast weight to move horizontally along the guide rail, thereby controlling the tilt of the octopus robot. The overall structure of the bionic octopus robot is designed to be vertically symmetrical. When the center of gravity adjustment device is in its initial position, the robot’s buoyancy center and center of gravity are aligned along the robot’s central axis, allowing the robot to remain in an upright position. As the ballast weight on the adjustment device is moved, the robot’s center of gravity shifts away from the central axis. This creates an imbalance between the torque generated by the buoyancy force and the torque generated by gravity, causing the robot to tilt until the center of buoyancy and center of gravity are aligned or the opposing torque is generated to achieve balance.

**Figure 5:**
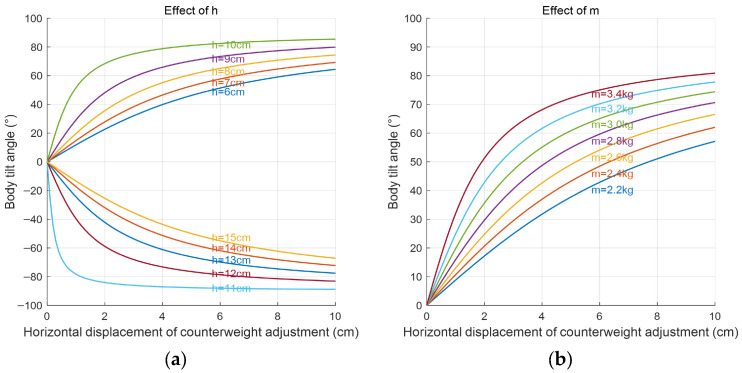
The effect of different heights and masses of the center-of-gravity adjustment device on the robot’s tilt angle. (**a**) Variation curves of tilt angles at different installation heights (upward tilting is positive, and downward tilting is negative). (**b**) Variation curves of tilt angles with different installation masses.

The trend of the robot’s tilt-angle variation curves at different heights is shown in Figure 5a, and the height is the CG adjustment device relative to a reference connection frame. For heights between 6 and 10 cm, the tilt-angle variation curves of the robot show a similar trend. As the horizontal displacement of the ballast weight on the center-of-gravity adjustment device increases, the robot’s body tilt angle initially increases rapidly; but then, the rate of change becomes more gradual. As the *h* parameter increases, this trend becomes more pronounced. At the same horizontal displacement of the ballast weight, a greater installation height results in a larger tilt angle of the robot. Therefore, the installation height of the robot’s center-of-gravity adjustment device is set to 8 cm. In addition to the installation height of the center-of-gravity adjustment device, the mass of the ballast weight also significantly affects the robot’s tilt-angle variation. Figure 5b shows the trend of the robot’s tilt-angle variation curves for different ballast weights. At a height of 8 cm, a larger ballast weight on the center-of-gravity adjustment device results in a steeper initial slope of the tilt-angle variation curve. Additionally, for the same horizontal displacement of the ballast weight, the robot’s tilt angle is greater. Considering the motor’s load capacity and the need for the center of buoyancy to be above the center of gravity, the ballast weight is chosen to be 2.6 kg. With the center-of-gravity adjustment device set at an installation height of 8 cm and a ballast weight of 2.6 kg, the theoretical maximum tilt angle of the biomimetic octopus robot can reach 68.5°.

## 3. Motion Mode of the Biomimetic Octopus Robot

### 3.1. Biomimetic Octopus Robot Walking Mode and Gait Planning

An octopus controls the movement of its two arms through the coordinated action of transverse, longitudinal, and oblique muscle bundles, enabling it to perform a bipedal walking motion. In addition to forward bipedal walking, an octopus can also move backward and occasionally use a third arm to drag or briefly support its weight. During bipedal walking, octopuses do not use a fixed pair of arms for walking. Instead, they may change which arms they use during movement, allowing them to move sideways [9]. Huffard [10] studied the speed and body-size changes of the shallow-water octopus (Abdopus aculeatus) during crawling, bipedal walking, swimming, and jet propulsion. The study found that the body mass of the octopus does not affect its walking speed. The average walking speed of the octopus was 1.34 ± 0.19 BL/s and 13.1 ± 11.5 cm/s, with a maximum speed of 2.25 BL/s and 19.0 cm/s. Compared to crawling, this mode of motion is faster and can be considered a rapid escape method that does not rely on jet propulsion.

**Figure 6:**
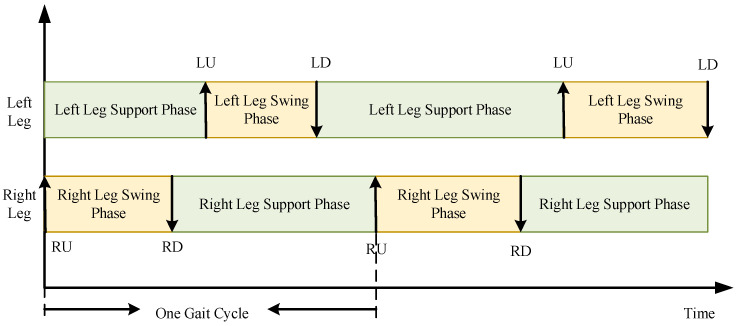
Simplified phase diagram of bipedal locomotion in the coconut octopus.

Researchers have found that during walking on the beach, an octopus’s gait phase is variable, with brief periods when no arms are in contact with the ground. However, on average, the stride length of each arm on the seabed exceeds half of its total reach. Figure 6 shows a simplified phase diagram of the bipedal walking gait of the coconut octopus [13]. In the diagram, L represents the octopus’s left walking arm, R represents the right arm, U indicates the arm being lifted off the seabed, and D indicates the arm being placed back onto the seabed. Based on the analysis of the octopus’s underwater bipedal walking, the arm movements can be divided into a support phase and a swing phase. During the support phase, the interaction forces between the tips of the arms and the seabed are transmitted to the body, creating a stepping motion. In the swing phase, the arms return to their original position for the next stance phase, generating a repetitive gait cycle.

**Figure 7:**
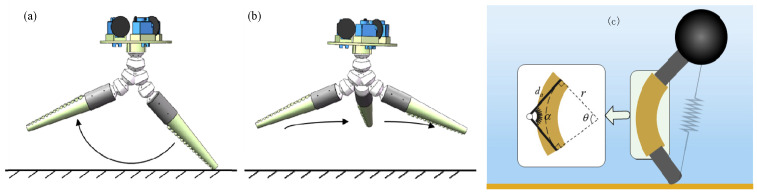
Schematic diagram of the support phase and swing phase of a single arm during the robot’s walking process. (**a**) Support phase. (**b**) Swing phase. (**c**) Transformation of the flexible actuator into a virtual hinge structure with torque springs [14].

Similarly, in the robot’s walking gait control process, the flexible drive arms are controlled separately in the support phase and the swing phase, as shown in Figure 7. This biomimetic robot differs from traditional quadruped robots with knee and hip joints and, therefore, cannot lift and return its legs within the same plane. Additionally, the legs cannot return along the same path, which increases the risk of the robot’s feet (tips of the walking arms) touching the ground and disrupting its dynamic stability during walking.

The plane angle rotates according to a linear rule: $\phi =\phi_{0}+\pi -\omega_{\phi }t$. When the curvature plane angle of the drive arm reaches its initial angle, the control cycle continues to repeat. All parameters and variables are listed in Table 1. 

The walking-gait expression of the biomimetic robot is described by the following equation:

Support Phase:

(1) ϕ=1−Cnπ+ϕ0α=α0+ωαt 

Swing Phase:

(2) ϕ=ϕ0+Cnπ−ωϕt+1−Cnωϕtα=α0 

When $C_{n}=1$, the walking arm swings in a clockwise direction from the robot’s top-down view. Otherwise, when $C_{n}=0$, the walking arm swings in a counterclockwise direction. $\omega_{\alpha }$ and $\omega_{\phi }$ are the rates of change of the virtual joint angle and curvature plane angle of the flexible drive arm, respectively. Their expressions can be described as follows:

(3) $$\omega_{\alpha }=\frac{2\pi -2\alpha_{0}}{T_{s}\times \beta }\;$$

(4) $$\omega_{\phi }=\frac{\pi }{T_{s}\times \left(1-\beta \right)}\;$$

Therefore, during the swing phase, a lateral return method is used, where the walking arm moves in a sideward arc back to the position of the next initial supporting leg, keeping the robot’s feet as far from the ground as possible.

The control cycle of a single arm in the biomimetic robot is divided into two phases: (1) Support Phase: First, the curvature plane angle of the flexible drive arm is kept constant to ensure the direction of the robot’s movement. Then, by controlling the virtual joint angle of the flexible drive arm, the arm swings backward according to the linear function expressed as $\alpha =\alpha_{0}+\omega_{\alpha }t$ once the robot reaches the touch-down condition. When the flexible drive arm reaches the maximum angle (arm reaches the maximum admissible curvature before cable saturation), it transitions into the swing phase. (2) Swing Phase: First, the curvature plane angle of the flexible drive arm symmetrically reverses, and the actuator returns to its initial curvature. During the return process, the actuator’s curvature angle remains constant.

Throughout the robot’s walking control process, the curvature plane angle and virtual joint angle of the flexible drive arm serve as gait output parameters. However, to control the flexible drive arm, it is still necessary to convert these two parameters into the lengths ($l_{i}$) of the three-line drive cables of the flexible drive arm:

(5) $$l_{i}=l-(\pi -\alpha )Rcos\left(\frac{2\pi }{3}\left(i-1\right)-\phi \right)\;$$

In the equation, R is the spacing radius of the three drive cables. The length *l* of the central line of the flexible drive arm is set as a constant throughout the control process, which is shorter than the initial length of the silicone arm and includes a certain amount of pre-tension.

### 3.2. Multi-Arm Swimming Mode of the Biomimetic Octopus Robot

#### 3.2.1. Multi-Arm Swimming Gait of the Biomimetic Octopus Robot

In order to explain the motivation of swimming gait design in this study, we briefly summarize the representative biological observation results and previous research on octopus-like robots. These studies revealed the common thrust generation principle (extension/contraction of tentacles and collaborative stroke) and emphasized the constraints faced by such robots in practical applications (driving bandwidth, limb compliance, and body stability), which directly provide the basis for the step parameterization proposed in this study.

Several different octopus swimming modes have been discovered [12]. (1) Jet Propulsion Swimming: The octopus uses its siphon while its arms are held closely together. (2) Head-First Swimming: The octopus swims with its siphon, with its head leading and its arms trailing and undulating. (3) Multi-Arm Swimming: The octopus synchronously moves its arms like opening and closing an umbrella, generating powerful propulsion and rapid acceleration.

Sfakiotakis et al. [7] recorded videos of an octopus’s multi-arm swimming, which involves synchronous paddling motion of all eight arms and consists of three distinct phases. In the first phase, the octopus’s arms are initially retracted; then, they open outward relatively slowly (recovery stroke). In the second phase, the arms quickly return to the initial position (power stroke), generating significant forward thrust. In the third phase, following the power stroke, the octopus’s arms remain closed, maintaining an inertial glide. This multi-arm swimming motion mode exhibits significant symmetry, and the movements are synchronized. The ratio of the duration of the recovery stroke to the power stroke was estimated to be approximately 2.5 ± 0.5. During a single multi-arm swimming event, the octopus can achieve a swimming speed of up to 88.42 mm/s.

**Figure 8:**
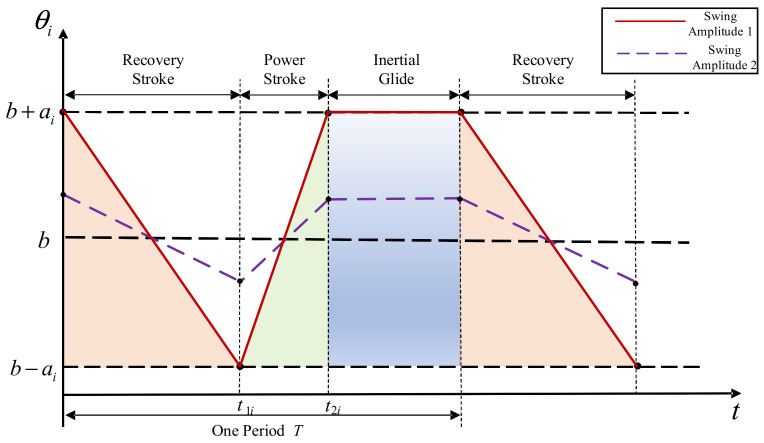
Swing angle of the swimming arms of the biomimetic octopus robot.

Previous studies have consistently shown that the thrust can be enhanced through the coordinated contact stroke with an appropriate phase relationship and that steering can be achieved by adjusting the asymmetric amplitude or phase between the contact angles. These research insights were accurately translated into the parameter settings in our gait controller (as shown in Table 2). Based on the motion characteristics of octopus multi-arm swimming, this paper divides the biomimetic robot’s multi-arm swimming mode into three stages: power stroke, recovery stroke, and inertial glide. The swing angle of the swimming arms during the multi-arm swimming process of the robot is illustrated in Figure 8. According to the figure, the single-arm stroke-motion equation for one cycle of the biomimetic robot can be described as follows:

(6) $$\theta_{i}=\left\{\begin{array}{cc} b+a_{i}-k_{1i}t & 0\leq t<t_{1i}\\ b-a_{i}+k_{2i}t & t_{1i}\leq t<t_{2i}\\ b+a_{i} & t_{2i}\leq t<T \end{array}\right.\;$$

In the equation, i=1,2,3,4, $\theta_{i}$ is represents the servo rotation angle of the *i*-th arm of the robotic octopus ($\theta_{i}$ refers to the servo/root joint angle; during experiments, $\theta_{i}$ was recorded as the commanded servo angle sent by the controller, i.e., the PWM command mapped to the angle via servo calibration), $a_{i}$ denotes the swing amplitude of the swimming arm, $k_{1i}$ is the opening speed of the swimming arm during the recovery stroke, and $k_{2i}$ is the closing speed of the swimming arm during the power stroke. By default, b is the midpoint value between the maximum swing angle ($\theta_{max}$) and the minimum swing angle ($\theta_{min}$) of the biomimetic robot’s swimming arm.

(7) $$b=\frac{\theta_{max}+\theta_{min}}{2}\;$$

In the equation, due to the structural constraints of the biomimetic robot, the maximum swing angle ($\theta_{max}$) and the minimum swing angle ($\theta_{min}$) are 0° and 90°, respectively.

The swimming arms of the biomimetic octopus robot are driven by Hitec HS-5086WP waterproof medium-sized digital metal servos with a working voltage of 6 V and a maximum rotation speed of $\omega_{max}=0.18\;s/60{}^{\circ}$. Therefore, during the power-stroke and recovery-stroke phase, the rotation speed of the swimming arms should be less than the maximum rotation speed of the servos, i.e., ${k_{1i}\leq \omega }_{max}$ and ${k_{2i}\leq \omega }_{max}$.

Based on the swinging and stationary states of the swimming arms, the entire swimming cycle can be further divided into the swinging time ($t_{mi}$) and the stationary time ($t_{si}$). The swinging time includes both the robot’s power stroke and recovery stroke, while the stationary time corresponds to the robot’s gliding process. For convenience in control, this paper uses the following parameters for the multi-arm swimming control of the robot: the speed ratio of the power stroke to the recovery stroke ($c_{i}=k_{2i}/k_{1i}$), the intermediate swing angle (b), the swing amplitude ($a_{i}$), the swinging time ($t_{mi}$), and the stationary time ($t_{si}$). Substituting the above parameters yields the following:

(8) $$k_{1i}=\frac{2a_{i}\left(1+c_{i}\right)}{c_{i}t_{mi}}\;$$

(9) $$k_{2i}=\frac{2a_{i}\left(1+c_{i}\right)}{t_{mi}}\;$$

Thus, the single-arm stroke motion equation for one cycle can be described as follows:

(10) $$\theta_{i}=\left\{\begin{array}{cc} b+a_{i}-\frac{2a_{i}\left(1+c_{i}\right)}{c_{i}t_{mi}}t & 0\leq t<\frac{c_{i}t_{mi}}{1+c_{i}}\\ b-a_{i}+\frac{2a_{i}\left(1+c_{i}\right)}{t_{mi}}t & \frac{c_{i}t_{mi}}{1+c_{i}}\leq t<t_{mi}\\ b+a_{i} & t_{mi}\leq t<t_{mi}+t_{si} \end{array}\right.\;$$

In this paper, the multi-arm swimming mode of the biomimetic robot is a synchronous stroking swimming pattern. Therefore, the swinging and stationary time of the four swimming arms should be kept consistent:

(11) $$t_{m1}=t_{m2}=t_{m3}=t_{m4}\;$$

(12) $$t_{s1}=t_{s2}=t_{s3}=t_{s4}\;$$

#### 3.2.2. Dynamics Model of the Robot’s Multi-Arm Swimming

Assuming the biomimetic robot is moving in a stationary fluid, is subjected to several forces during swimming: the thrust force generated by the swinging of its swimming arms, the drag force exerted by the fluid on the robot, the inertial force during acceleration, and the reaction force from the surrounding fluid due to the robot’s acceleration or deceleration. The force balance equation for the biomimetic robot can be expressed as follows:

(13) T=F+G+D 

In the equation, *T* is the thrust, *F* is the inertial force, *G* is the acceleration reaction force, and *D* is the drag force exerted by the water on the robot’s body.

In swimming mode, the thrust of the biomimetic octopus robot is primarily generated by its swimming arms. By exploiting the resistive properties of water, the robot’s swimming arms perform a backward stroke, exerting force on the water. This action transfers momentum to the water, changing the momentum of the water particles. Consequently, the robot experiences a reactive force from the water that propels it forward.

**Figure 9:**
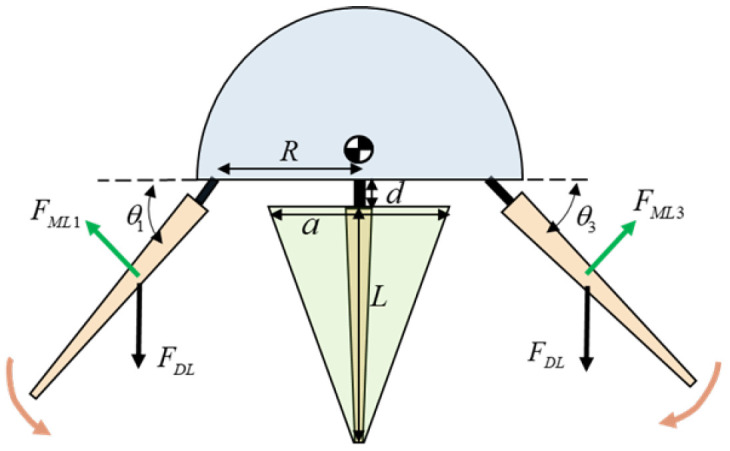
Force analysis of the swimming arms of the biomimetic octopus robot.

The single swimming arm of the robot and the membrane between the arms form an inverted triangle, as shown in Figure 9. The relationship for the relative width of the membrane is expressed as follows:

(14) $$W_{leg}\left(l\right)=a\left(1-\frac{l}{L}\right)\;$$

In the equation, a is the width of the membrane between the arms at the base, L is the length of the swimming arm, and l is the relative position of the swimming arm.

The fluid resistance model [15] is used to analyze the fluid interaction of the swimming arms of the biomimetic octopus robot. The thrust force ($F_{MLi}$) generated by a single arm interacting with the surrounding fluid can be expressed as follows:

(15) $$F_{MLi}=\frac{\rho }{2}C_{dL}\left|{\dot{\theta }}_{i}\right|{\dot{\theta }}_{i}\int_{0}^{L}{\left(d+l\right)}^{2}W_{leg}\left(l\right)dl$$

The leg-drag coefficient is $C_{dL}$, and the direction of this force is opposite to the swinging velocity direction of the swimming arm. $\theta_{i}$ is the angle between the swimming arm and the robot’s chassis.

We assume that the mass of the swimming arms of the robotic octopus is negligible compared to the mass of the head and the robot’s center of mass is located at the geometric center of the torso.

When the robot operates in a synchronous multi-arm stroking mode, some forces between the different swimming arms partially cancel each other out. Therefore, the total thrust force *T* of the robotic octopus can be simplified as follows:

(16) $$T=\sum_{i=1}^{4}F_{MLi}cos\theta_{i}\;$$

To simplify the complexity of the model, the biomimetic robot is assumed to have a bell-shaped morphology [16]. The acceleration reaction force and inertial force experienced by the body can be expressed as follows:

(17) $$G=\alpha \rho V\dot{v}\;$$

(18) $$F=m\dot{v}\;$$

In the equation, *α* is the added mass coefficient and *V* is the volume of the bell-shaped body of the robot, which can be expressed in terms of the diameter and height of the bell shape as follows:

(19) $$\alpha ={\left(2\left(R+Lsin\theta \right)/\left(2R+2Lcos\theta \right)\right)}^{1.4}\;$$

(20) $$V=\frac{1}{3}\pi R^{3}+\frac{1}{3}\pi L(R^{2}+Rr_{1}+{r_{1}}^{2})\;$$

where $r_{1}$ is the radius of the base of the bell shape, which can be expressed as $r_{1}=R+Lcos\theta$.

During swimming, the robot experiences drag forces from both the head compartment and the swimming arms. The drag force can be expressed as follows:

(21) $$D=D_{h}+{4D}_{Li}\;$$

(22) $$D_{h}=0.5C_{dh}\rho \pi R^{2}v^{2}\left(t\right)\;$$

(23) $$D_{Li}=0.25C_{dL}\rho aLcos\theta_{i}v^{2}\left(t\right)\;$$

In the equation, $D_{h}$ and $C_{dh}$ are the drag force and drag coefficient on the robot’s head compartment, respectively, while $D_{Li}$ is the drag force on a single arm. It is assumed that the drag coefficient for the head compartment is the same as that for a hemisphere, with $C_{dh}=0.42$.

## 4. Underwater Motion Experiments of the Robot

### 4.1. Control System and Experimental Setup

The proposed robot is driven by the embedded control system to ensure a completely untethered experiment. A 32-bit microcontroller is built into the sealed head cabin to execute the gait generation algorithm of walking and swimming modes. The cable-driven walking arm is driven by a DC motor with an encoder through a custom driver, while the swimming arm is driven by a waterproof digital actuator. The main controller communicates with the driving unit through a robust serial bus. At the same time, the 2.4 GHz wireless link is used for parameter optimization and data recording. The depth sensor and inertial measurement unit (IMU) provide buoyancy and attitude feedback for setting of the working point of buoyancy adjustment and gravity balance device. In all experiments, the predefined open-loop gait contour performed by the vehicle was used, and the robot trajectory was recorded by the upper camera for offline quantitative analysis so as to realize the repeatability evaluation of motion performance.

### 4.2. Underwater Straight-Line Walking Experiments of the Robot

To verify the biomimetic robot’s underwater straight-line walking capability and the driving performance of the flexible drive arms, underwater straight-line walking experiments were conducted in this section. An optical camera was used to record the robot’s motion in real time, and the recorded data was analyzed using Tracker software (version 5.x) to obtain the trajectory of the robot’s center of mass.

**Figure 10:**
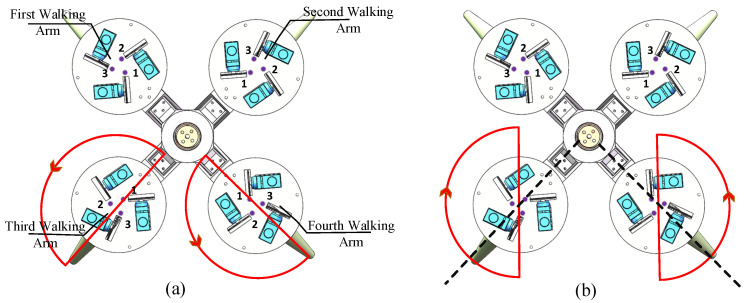
Swing trajectory of the robot’s walking arm. (**a**) Default parameter gait trajectory. (**b**) Straight-line walking gait trajectory.

During the bipedal walking experiments, the biomimetic robot used the third and the fourth arms for underwater bipedal walking. Figure 10a shows the swinging motion of the walking arms of the robot’s default parameters ($\phi_{0}=0{}^{\circ},\alpha_{0}=90{}^{\circ},C_{n}=0$). In Figure 10, the red line represents the swing trajectory of the end of the walking arm from a top-down view, and numbers 1–3 indicate the installation sequence of drive cables 1–3. According to the observations in Figure 10, under the default parameters, the swinging trajectory of the walking arm is counterclockwise and located to the left of the symmetric dashed line. To enable the robot to achieve straight-line walking underwater, the swinging direction and initial bending plane angle of the walking arms need to be adjusted. Therefore, the parameters of the third arm were adjusted to $\phi_{0}=45{}^{\circ},C_{n}=1$, while the parameters for the fourth arm were adjusted to $\phi_{0}=135{}^{\circ},C_{n}=0$. The swinging trajectory of the walking arms after these adjustments is shown in Figure 10b.

**Figure 11:**
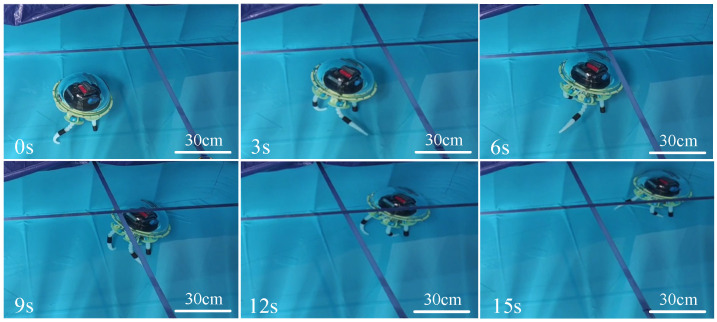
Underwater straight-line walking experiment of the biomimetic robot.

To facilitate the observation of the robot’s walking arm motion, the swimming arms and the first two walking arms were not installed during the underwater straight-line walking experiment, control parameter settings for the robot’s straight-line walking gait in Table 2. Additionally, to ensure that the robot was fully submerged, the buoyancy device was filled with water, adjusting the robot’s weight in the water to 50 g. Figure 11 and Supplementary Materials shows the process of the test experiment of the robot’s bipedal walking. During the experiment, the robot exhibited a generally smooth walking trend, and the alternating motion between the support phase and the swing phase of the walking arms was clearly observable. This observation validates the feasibility of the structural design and walking gait of the biomimetic octopus robot during underwater walking movements. Through multiple experiments, the robot achieved an average speed of 7.26 cm/s during straight-line walking. However, removing the front swimming arms and the first two walking arms changes the hydrodynamic drag and the mass/buoyancy distribution of the system, which may affect the required ground contact force and the overall walking stability. With the full eight-arm configuration, additional drag and altered weight distribution may slightly reduce the walking speed and require re-tuning of the ballast/CG settings. The robot experienced multiple instances of slipping during walking. This issue may be due to the relatively light weight of the biomimetic robot underwater, which reduces the friction between the walking arms and the pool floor. To address this issue, future work could consider integrating active suction cups into the walking arms. By using controllable suction cups, the robot can achieve single-arm adhesion to the ground, thereby reducing slipping during walking. This improvement could increase the contact force between the robot and the pool floor, enhancing walking stability and accuracy.

### 4.3. Underwater Steering and Walking Experiments of the Robot

**Figure 12:**
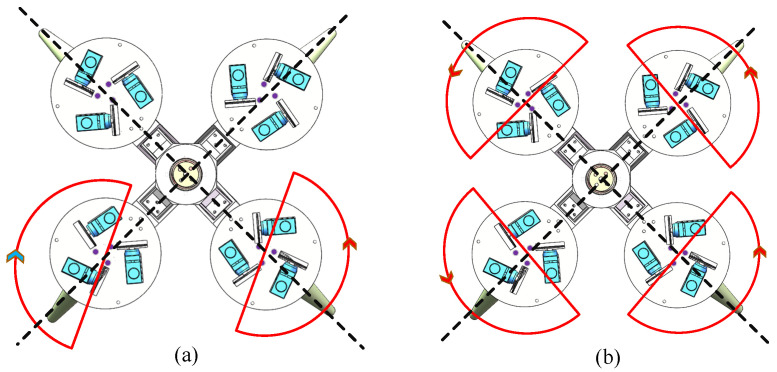
Swing trajectory of the walking arms during underwater turning of the robot. (**a**) Forward movement and turning. (**b**) In-place straight turn.

Due to the various combinations and choices of parameter settings in the walking gait and the activation of the four walking arms, the robot’s walking modes are diversified. The implementation of underwater forward movement and turning is achieved by modifying the initial bending-plane angle between two walking arms based on the underwater straight-line locomotion, as shown in Figure 12a.

**Figure 13:**
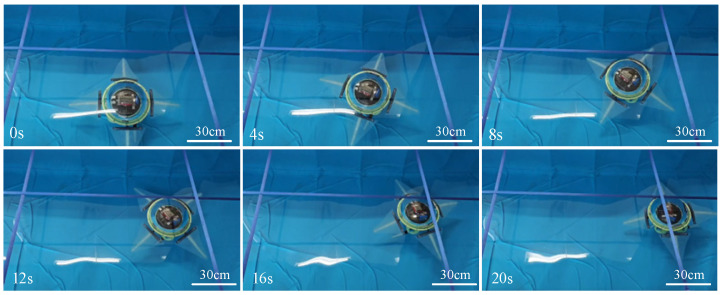
Underwater forward rightward turning walking experiment of the biomimetic robot.

Compared to the control of the robot’s underwater straight-line walking gait, the gait parameters for a right-turning walking arm or left-turning walking arm while moving forward are similar to those for straight-line walking. However, it is necessary to modify the initial bending-plane angle parameters of the two walking arms to change the angle of the supporting phase during walking. This allows the robot to achieve angular deviation during walking, thereby enabling it to turn while walking. Therefore, based on the gait parameters from the previous section on underwater straight-line walking experiments, the initial bending-plane angle parameters of the two walking arms are set to 25° and 15°, respectively (with both arms deviating 20° counterclockwise from straight-line walking parameters, i.e., $\phi_{0}$). An experiment was conducted to test the robot’s rightward turning while moving forward. Figure 13 shows the robot’s motion during the underwater forward rightward turning experiment.

Additionally, the robot’s in-place turning motion requires all four walking arms to be activated simultaneously, with their initial bending plane angle set to $\phi_{0}=90{}^{\circ}$. The direction of the in-place rotation can be controlled by adjusting the $C_{n}$ parameters of the four walking arms. A schematic of the robot’s swinging trajectory for counterclockwise in-place turning is shown in Figure 12b. The experimental parameters for the robot’s counterclockwise in-place turning are set as shown in Table 3, where the gait parameters for the four walking arms are the same during the in-place turning experiment.

**Figure 14:**
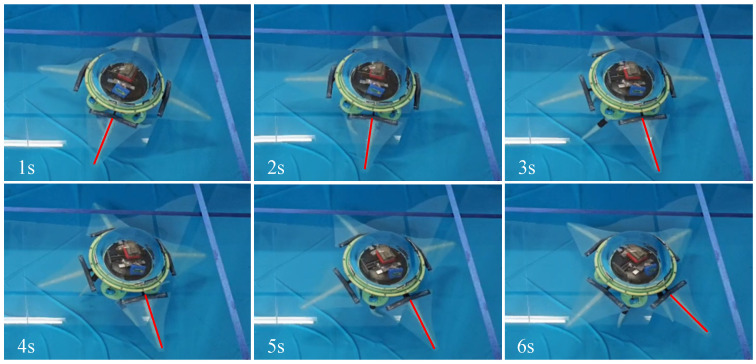
Underwater counterclockwise in-place turning experiment of the biomimetic robot.

Based on the gait control parameters from Table 3, the robot was placed in the pool for in-place turning experiments. Figure 14 shows the motion state of the biomimetic robot during counterclockwise in-place turning over two motion cycles. The robot took 6 s to complete two cycles, turning a total of 74.6°. On average, the robot could turn 37.3° per cycle.

### 4.4. Multi-Arm Straight-Line Swimming Experiment of the Robot

To evaluate the straight multi-arm swimming performance of the biomimetic octopus robot, we conducted a straight multi-arm swimming experiment in a pool. The following experimental parameters were set for the robot: thrust velocity, velocity ratio, gliding time, intermediate swing angle, and swing amplitude. By controlling the buoyancy adjustment device, we ensured that the density of the robot was slightly lower than that of water, thereby preventing it from sinking and maintaining a near-neutral buoyancy state on the water’s surface. Additionally, the center-of-gravity adjustment device was utilized to tilt the robot to its maximum angle. An optical camera was fixed above the pool to record the horizontal displacement of the robot in real time.

**Figure 15:**
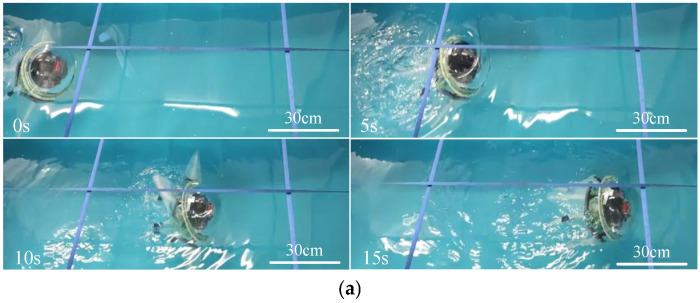

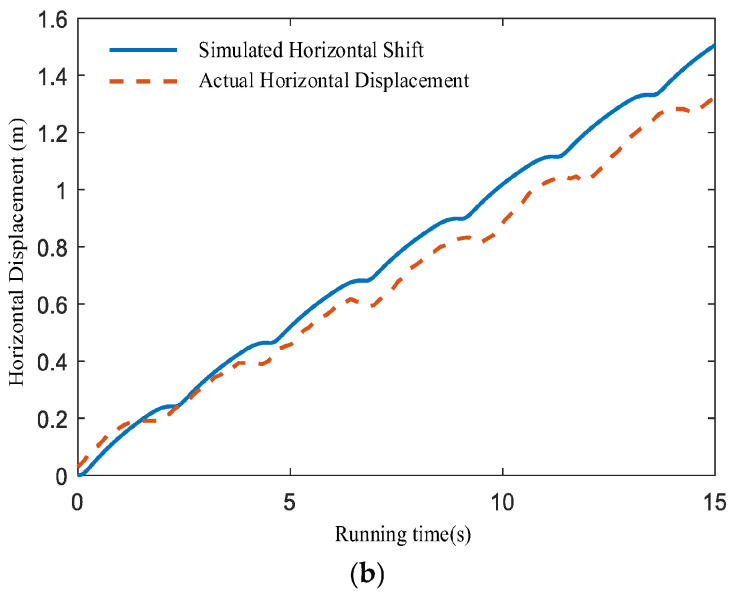
Straight-line multi-armed swimming of the biomimetic octopus robot. (**a**) Screenshots from straight-line swimming videos. (**b**) Experimental vs. simulation results for bionic octopus multi-arm swimming.

Figure 15 shows the process of the multi-arm swimming experiment. The total duration of the swimming test was 15 s, during which the robot traveled a distance of 1.3 m, with an average speed of 8.6 cm/s and a maximum instantaneous speed of 15.1 cm/s. During multi-arm swimming, the propulsion strokes generated significant forward thrust, causing the robot to accelerate noticeably. Conversely, during the recovery phase, backward thrust was generated, resulting in noticeable deceleration or backward movement of the robot. The overall motion trends of the experiment and simulation are similar, but there are still differences in numerical values. The average swimming speed of the robot in the experiment was slightly lower than the simulation data. This discrepancy can be attributed to two potential reasons. First, the use of flexible silicone in the swimming arms causes the arms to bend during swimming, reducing the thrust area and resulting in a slight decrease in speed compared to the simulation. Second, during the testing process, as the robot floated on the water surface, one of the swimming arms occasionally emerged from the water while being pushed, which reduced the generated thrust.

### 4.5. Turning Multi-Arm Swimming Experiments of the Robot

**Figure 16:**
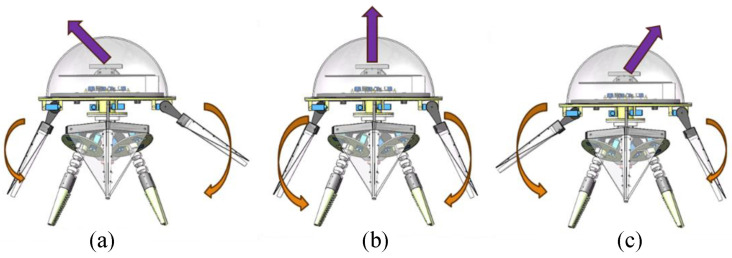
Schematic diagram of the biomimetic robot’s turning swimming. (**a**) Swimming to the left. (**b**) Swimming forward. (**c**) Swimming to the right.

Various asymmetric movements of the robot’s walking arms can alter the overall direction of the system, resulting in a range of ‘turning gaits’. According to the analysis of gait parameters, the swing amplitude *a* and speed ratio *c* are the key parameters affecting the thrust and speed of the biomimetic robot. In theory, by setting different parameters for the robot’s left and right walking arms, asymmetric movements can be achieved, enabling the biomimetic robot to perform turning swimming. However, setting different speed-ratio *c* parameters can cause the propulsion and recovery stroke cycles of the two arms to overlap, leading to a lack of coordination in the robot’s multi-arm swimming mode. To address this, the swing amplitude *a* was selected as the primary control parameter for the turning gait. For example, setting a smaller swing amplitude for the left swimming arm compared to the right swimming arm causes the robot to turn left while swimming and vice versa when turning right, as shown in Figure 16.

**Figure 17:**
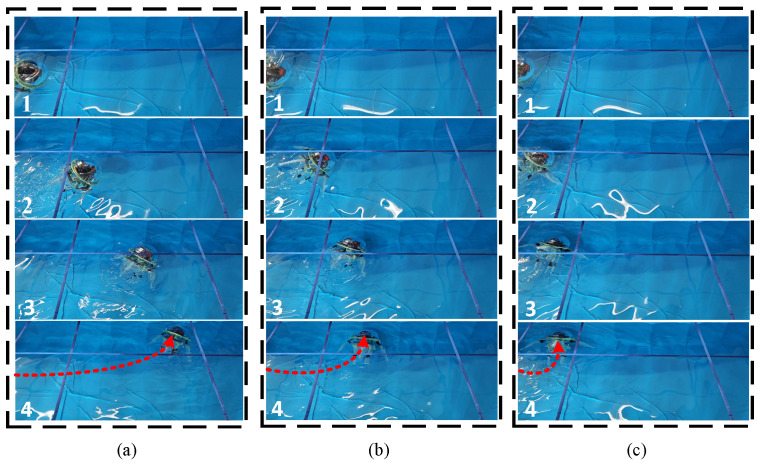
Video snapshots of the robot’s leftward swimming with different swing amplitude parameters. (**a**) Left-arm swing amplitude of 40°. (**b**) Left-arm swing amplitude of 30°. (**c**) Left-arm swing amplitude of 20°.

To analyze the effect of this turning gait on the biomimetic robot, three different gait parameters were selected for experimental comparison. During the experiments, the parameters for the biomimetic robot’s turning gait were kept consistent with those for the straight-line gait, except for the swing amplitude parameter of the left arm. Figure 17 shows the experimental motion states of the biomimetic robot under three different swing amplitude conditions for the left arm.

**Figure 18:**
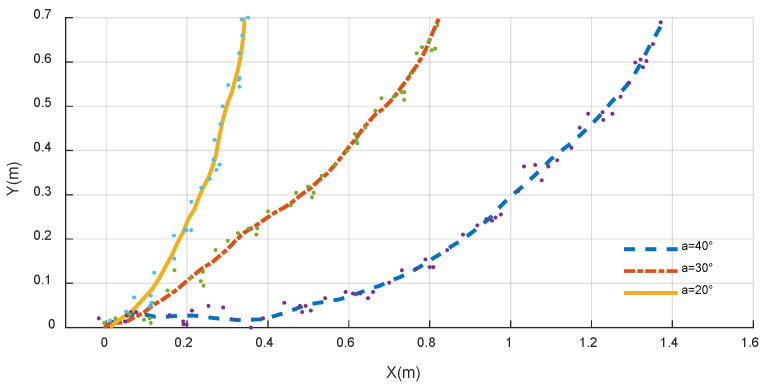
Top-down view of the robot’s center-of-mass trajectory under different turning gait parameters.

Under three different parameter settings, the biomimetic robot started from the midpoint of the left wall of the pool and ended when it struck the back wall of the pool. Figure 18 shows the top-down view of the underwater left-turning swimming trajectory of the biomimetic robot. For left-arm swing amplitudes of 40°, 30°, and 20°, the corresponding average turning angular velocities of the robot were 3.67°/s, 5.59°/s, and 10.28°/s, respectively, and the average speeds were 8.22 cm/s, 7.84 cm/s, and 7.31 cm/s. The robot’s average speed decreases nonlinearly as the left arm’s swing amplitude decreases, while the average angular turning velocity increases as the left arm’s swing amplitude decreases.

### 4.6. Thrust-Force Experiments of the Biomimetic Octopus Robot

**Figure 19:**
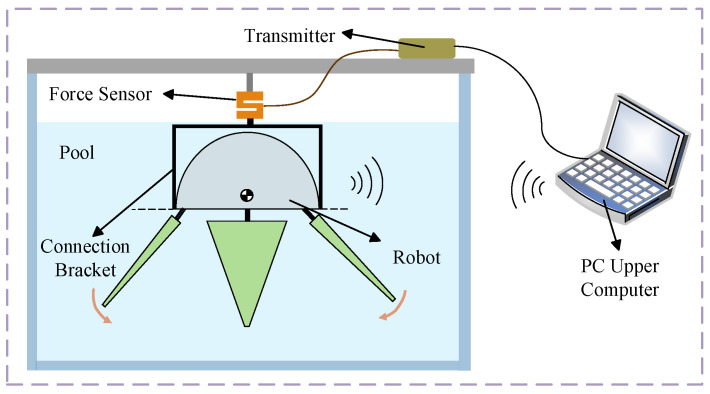
Schematic of the thrust-force test platform.

Additionally, in this section, thrust-force performance tests of the robot were conducted using a thrust-force testing platform, as shown in Figure 19.

The thrust-force testing platform consists of two parts: the thrust acquisition system and the motion control system. The motion control system uses monitoring software to communicate with and control the biomimetic robot via a wireless model. The thrust-force acquisition system primarily amplifies the analog signals output by the force sensors, then collects and transmits these signals to the upper computer on the PC. It consists of three components: the force sensor, the transmitter, and the signal collector. Due to the robot’s center-of-gravity adjustment device being unable to achieve a completely horizontal orientation, during thrust-force testing, the biomimetic robot was maintained in a vertical position and submerged in water and was fixed in the pool using a connection bracket.

**Figure 20:**
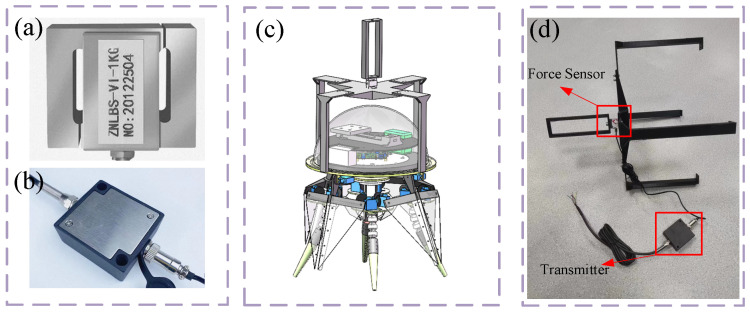
Thrust-force testing platform of the biomimetic robot. (**a**) Force sensor, (**b**) Transmitter, (**c**) Protype of thrust-force test platform, (**d**) Support frame of test platform.

Based on the maximum thrust-force calculated from the simulation, an S-type tension sensor (model ZNLBS-VI-5KG) was selected, with dimensions of 30.0 mm × 13.6 mm × 25.0 mm, as shown in Figure 20a. The sensor has a measurement range of −50 N to 50 N and an IP67 waterproof rating. Due to the weakness of the output signal from the force sensor, a compatible transmitter (type: BSQ-3; input: mV; output: 0–5 V) was used to amplify the signal, as shown in Figure 20b. The amplified signal was then transmitted to the upper computer via the sampling ADC module and Rs232 serial port of the STM32 MCU. The voltage data was converted into pressure data based on the sensor’s scaling factor and displayed as a curve. The upper computer software was developed using QT.

**Figure 21:**
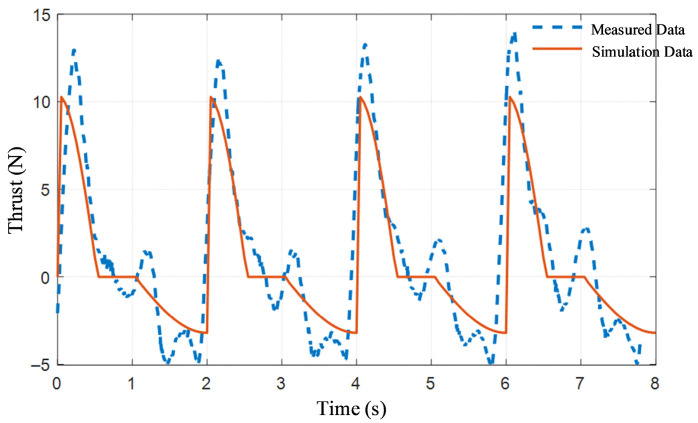
Comparison of thrust simulation results and experimental data.

To validate the effectiveness of the simulation results, a comparison was performed between the simulation results and experimental data for specific motion states. The dynamics of multi-arm swimming was simulated by MATLAB (version 2023a). In each time step, the instantaneous arm angle was calculated by using the preset joint trajectory of the swimming arm according to Equation (10), and the effective projected area and relative velocity were obtained accordingly. Then, the hydrodynamic thrust, resistance, and additional mass force were calculated using Equations (15)–(23) and integrated in a stroke cycle to estimate the net thrust and body speed. The model parameters (including resistance coefficient and added mass coefficient) were selected from the existing data of bell-shaped organisms and were verified by sensitivity analysis. The comparison curve of thrust-force testing experimental data and simulation results under the conditions of a swing amplitude of a=45°, intermediate swing angle of b=45°, speed ratio of c=2.0, swing time of $t_{m}=1.5\;s$, and gliding time of $t_{s}=0.5\;s$ is shown in Figure 21. As shown in Figure 21, the trend of the thrust-force simulation data is generally consistent with the experimental test data over time. The peak thrust force of the experimental data is slightly higher than that of the simulation data, with a maximum peak thrust force of 14.1 N. However, there are some fluctuations during the gliding and recovery phases, with significant fluctuations mainly occurring right after the end of the power stroke.

The deviations between the simulation results and the experimental data may be attributed to two possible reasons. First, the simulation is based on a simplified drag model and does not account for turbulence and fluid separation in the actual measurements, which results in a slightly higher measured thrust force compared to the simulated value. Secondly, the shallow depth of the pool and its proximity to the water surface may cause significant disturbances. After the power stroke, the water flow generated by the swimming arm can lead to reflections from the pool’s bottom and fluctuations on the water surface, resulting in larger fluctuations in the thrust-force measurements during the gliding and recovery phases.

### 4.7. Discussion and Comparison with Existing Octopus-Inspired Robots

To position the proposed design within the literature, a concise comparison with representative octopus-inspired robots is summarized in Table 4. Compared with PoseiDRONE, the soft eight-arm OCTOPUS robot, Kraken, and the multi-material Octobot, the robot presented herein uniquely integrates bipedal walking and synchronous multi-arm swimming in a fully self-contained platform with active buoyancy and center-of-gravity control, enabling controllable straight-line motion and turning in both modes. Specifically, PoseiDRONE, the OCTOPUS robot, Kraken [12], and the multi-material octopus swimmer [17,18] are taken as representative benchmarks for octopus-inspired soft underwater platforms.

## 5. Conclusions

This study presented a novel octopus-inspired soft underwater robot capable of both bipedal walking and multi-armed swimming. The robot adopts a rigid–flexible hybrid structure, composed of a central capsule and eight cable-driven soft arms, and integrates a buoyancy regulation system and a center-of-gravity adjustment mechanism to maintain postural stability across different locomotion modes.

Based on the motion principles of octopus bipedal walking and multi-armed swimming, gait generation strategies were developed for both walking and swimming. Experimental results demonstrated that the robot can perform straight-line walking, turning, and in-place rotation with an average walking speed of 7.2 cm/s. In swimming mode, the robot achieved a peak thrust of 14.1 N, an average swimming speed of 8.6 cm/s, and a maximum speed of 15.1 cm/s, validating the effectiveness of the proposed mechanical design and gait control.

Overall, the main contributions of this work include the following:

(1) The design of an octopus-inspired rigid–flexible hybrid underwater soft robot integrating dual locomotion modes;
(2) The development of bio-inspired gait generation strategies enabling stable walking and agile swimming; and
(3) The experimental validation of thrust performance and multi-modal locomotion capability.

Compared with PoseiDRONE and other existing Octopus-like systems, the robot proposed in this study realizes fully autonomous operation, flexible direction control, and low environmental disturbance and does not need external power support. The design lays a feasible foundation for the development of an adaptive and multifunctional underwater robot that can perform tasks in an unstructured marine environment. Future work will focus on enhancing autonomous control, as well as perception fusion and energy optimization, and explore its potential applications in underwater patrol, environmental monitoring, and unexploded ordnance (UXO) removal. Subsequent research will focus on the following aspects: closed-loop gait adaptation based on perception, the integration of active suction cups to suppress slip during walking, adaptive switching between walking and swimming modes according to terrain and flow-field conditions, and long-term pool and outfield experiments in real marine environments.

## Figures and Tables

**Table 1:** Parameters of the walking gait.

Parameter Symbol	Parameter Name	Parameter Symbol	Parameter Name
ϕ (°)	Curvature plane angle (Figure 2b)	β	Proportion of the support phase in the gait cycle
$\phi_{0}\;$(°)	Initial curvature plane angle	$C_{n}$	Gait swing direction (clockwise/counterclockwise)
α (°)	Virtual joint angle	$\omega_{\alpha }$	Rate of change of the virtual joint angle
$\alpha_{0}$ (°)	Initial virtual joint angle	$\omega_{\phi }$	Rate of change of the curvature plane angle
$T_{s}$ (s)	Gait cycle	on_off	Whether to enable the flexible arm

**Table 2:** Control parameter settings for the robot’s straight-line walking gait.

Parameter Name	Parameter Symbol	Third Walking Arm	Fourth Walking Arm
Ratio of the support phase in the gait cycle	β	0.6	0.6
Initial virtual joint angle (°)	$\alpha_{0}$	90°	90°
Initial bending-plane angle (°)	$\phi_{0}$	45°	135°
Gait cycle period (s)	$T_{s}$	3 s	3 s
Gait swing direction (clockwise/counterclockwise)	$C_{n}$	0	1

**Table 3:** Control parameters for the counterclockwise turning gait of the robot.

Parameter Name	Parameter Symbol	Parameter Value
Ratio of the supporting phase in the gait cycle	β	0.6
Initial virtual joint angle	$\alpha_{0}$	60°
Initial bending-plane angle	$\phi_{0}$	90°
Gait cycle duration	$T_{s}$	3 s
Gait swing direction (clockwise/counterclockwise)	$C_{n}$	1

**Table 4:** Comparison with representative octopus-inspired robots.

Robot	Locomotion Modes	Power/Control Architecture	Representative Features/Performance
PoseiDRONE	Crawling, jet-based swimming, and manipulation	Tethered or externally powered soft-bodied ROV	Soft-bodied ROV and limited autonomy
OCTOPUS robot	Underwater walking and grasping	Partially tethered hybrid system with soft arms	Demonstrated multi-arm soft walking and grasping, with
Kraken	Swimming and grasping	Wireless control with hybrid actuators	emphasis on grasping
Octobot	Synchronous multi-arm swimming	Externally supplied or dedicated actuation	Soft tentacles and swimming at ~2.5 cm/s
This work	Bipedal walking and multi-arm synchronous swimming	Fully untethered platform with on-board integrated control, cable-driven limbs, and active buoyancy/CG regulation	Walking at 7.2 cm/s, swimming up to 15.1 cm/s, and thrust up to 14.1 N

## Data Availability

The original contributions presented in this study are included in the article/Supplementary Materials. Further inquiries can be directed to the corresponding author.

## Supplementary Materials

The following supporting information can be downloaded at: https://www.mdpi.com/article/10.3390/biomimetics11010059/s1, Video S1: bipedal walking; Video S2, Video S3, Video S4: multi arms swimming.
